# Modeling the impact of prevention policies on future diabetes prevalence in the United States: 2010–2030

**DOI:** 10.1186/1478-7954-11-18

**Published:** 2013-09-18

**Authors:** Edward W Gregg, James P Boyle, Theodore J Thompson, Lawrence E Barker, Ann L Albright, David F Williamson

**Affiliations:** 1 Division of Diabetes Translation, National Center for Chronic Disease Prevention and Health Promotion, Centers for Disease Control and Prevention, Atlanta, GA 30341, USA

## Abstract

**Background:**

Although diabetes is one of the most costly and rapidly increasing serious chronic diseases worldwide, the optimal mix of strategies to reduce diabetes prevalence has not been determined.

**Methods:**

Using a dynamic model that incorporates national data on diabetes prevalence and incidence, migration, mortality rates, and intervention effectiveness, we project the effect of five hypothetical prevention policies on future US diabetes rates through 2030: 1) no diabetes prevention strategy; 2) a “high-risk” strategy, wherein adults with both impaired fasting glucose (IFG) (fasting plasma glucose of 100–124 mg/dl) and impaired glucose tolerance (IGT) (2-hour post-load glucose of 141–199 mg/dl) receive structured lifestyle intervention; 3) a “moderate-risk” strategy, wherein only adults with IFG are offered structured lifestyle intervention; 4) a “population-wide” strategy, in which the entire population is exposed to broad risk reduction policies; and 5) a “combined” strategy, involving both the moderate-risk and population-wide strategies. We assumed that the moderate- and high-risk strategies reduce the annual diabetes incidence rate in the targeted subpopulations by 12.5% through 2030 and that the population-wide approach would reduce the projected annual diabetes incidence rate by 2% in the entire US population.

**Results:**

We project that by the year 2030, the combined strategy would prevent 4.6 million incident cases and 3.6 million prevalent cases, attenuating the increase in diabetes prevalence by 14%. The moderate-risk approach is projected to prevent 4.0 million incident cases, 3.1 million prevalent cases, attenuating the increase in prevalence by 12%. The high-risk and population approaches attenuate the projected prevalence increases by 5% and 3%, respectively. Even if the most effective strategy is implemented (the combined strategy), our projections indicate that the diabetes prevalence rate would increase by about 65% over the 23 years (i.e., from 12.9% in 2010 to 21.3% in 2030).

**Conclusions:**

While implementation of appropriate diabetes prevention strategies may slow the rate of increase of the prevalence of diabetes among US adults through 2030, the US diabetes prevalence rate is likely to increase dramatically over the next 20 years. Demand for health care services for people with diabetes complications and diabetes-related disability will continue to grow, and these services will need to be strengthened along with primary diabetes prevention efforts.

## Background

International surveillance data have documented a steady rise in diabetes prevalence over the past 40 years in virtually all regions of the world [1,2]. Although the increases in prevalence are, in part, due to increasing survival of persons with diabetes, national surveys have also described a doubling of the incidence of diagnosed diabetes over the past two decades [3,4]. These trends are concerning because of the well-documented deleterious effects of diabetes on mortality rates, vision loss, kidney disease, amputation, cardiovascular diseases, and disability [5,6]. Despite these trends, major randomized controlled trials conducted in diverse populations around the world now provide encouraging evidence that diabetes is preventable [7]. Specifically, among persons with impaired glucose tolerance (IGT), intensive lifestyle intervention reduces the incidence of type 2 diabetes by about 50%, while the drug metformin reduces the incidence by about 30% [8,9]. However, experts disagree on what policies would be most effective in reducing the long-term trends in the diabetes epidemic. Some have suggested that policies should focus on the entire population so as to make individuals’ default decisions healthy and require minimal individual effort [10,11], whereas others have argued for more intensive preventive interventions targeting higher-risk subpopulations [12]. Estimation of the potential impact of different strategies on diabetes prevalence ultimately requires information on prevalence, incidence, mortality, and intervention effectiveness that are not available in any single population study.

We are not aware of previous attempts to estimate the impact of different prevention strategies on future US diabetes prevalence rates. In these analyses, we used a dynamic model based on difference equations [13] and national diabetes prevalence, incidence, and mortality data to estimate the impact of population-targeted and intensive prevention programs on future diabetes prevalence.

## Methods

We constructed a multistate epidemiologic model with a time-varying transition matrix that divides the US adult population into five groups: those with normal glucose levels, those with impaired fasting glucose (IFG) (i.e., regardless of IGT status), those with both IFG and IGT, those with undiagnosed diabetes, and those with diagnosed diabetes. Using the model, we then estimated the impact of the following five hypothetical diabetes prevention strategies on projected diabetes prevalence rates among US adults through 2030: 1) no intervention (in which we assumed that current diabetes trends would continue unabated); 2) implementation of a national “high-risk” strategy, in which adults who have both IFG and IGT are eligible to participate in a structured lifestyle intervention; 3) implementation of a national “moderate-risk” strategy, in which adults with IFG receive a structured lifestyle intervention; 4) implementation of a “population-wide” strategy in which the entire US adult population is exposed to environmental and economic policies aimed at reducing risk factors for type 2 diabetes; and 5) implementation of a “combined” strategy, wherein population-wide strategies (strategy #4 above) are applied to the entire population while the moderate-risk strategy is simultaneously applied only to those who are identified as having IFG (strategy #3 above).

We assumed that the moderate- and high-risk strategies would result in a net 12.5% reduction in diabetes incidence in the targeted groups. This is based on a scenario in which 50% of the population is identified, 50% agree to participate, and one-year incidence is reduced by 50% among those who participate [14-16]. This assumption was based in part on findings from studies of hypertension control interventions [17] showing that roughly half of the target population is usually identified and that roughly half of those identified agree to participate in the interventions. We also conducted sensitivity analyses in which we assumed that the net reduction in the diabetes incidence rate would be half (i.e., 6.25%) and twice (i.e., 25%) of this base case assumption. We assumed that the population-wide intervention would lead to a 2% reduction in annual diabetes incidence in the entire US population. Because we had no data on the effectiveness of population-wide approaches for diabetes prevention, we based this assumption largely on the results of a meta-analysis of studies concerning reductions in cardiovascular disease (CVD) incidence associated with community interventions [17], which showed an average relative net decrease in predicted CVD incidence of 9.1% over an average follow-up period of 5.5 years (an average yearly net decrease of 1.8%). Other data we considered in estimating the effectiveness of a population-wide diabetes prevention intervention were results of the widely referenced North Karelia Project, which showed yearly net decreases in coronary mortality rates of 2.2% among women and 1.1% among men associated with community-wide cardiovascular disease risk reduction efforts [18]. In light of these findings, we assumed a 2% decline in our base case analyses, and again conducted sensitivity analyses in which we assumed that a population-wide intervention would reduce diabetes annual incidence rates by half (i.e., 1%) and twice (i.e., 4%) the amount that we assumed in our base case analyses.

The principal data sources for our dynamic model were the US Census Bureau, the National Diabetes Surveillance System, and published findings from selected epidemiologic studies and meta-analyses [13]. The model’s starting estimates of the prevalence of diagnosed diabetes, undiagnosed diabetes, IGT, and IFG among US adults aged 20 or older were based on 2005–2008 data from the National Health and Nutrition Examination Survey (NHANES) [19]. In NHANES, participants who reported having received a physician’s diagnosis of diabetes were classified as having “diagnosed diabetes”. Those without diagnosed diabetes but with a fasting plasma glucose (FPG) level ≥126 mg/dl or a two-hour postchallenge glucose level ≥200 mg/dl were classified as having undiagnosed diabetes [20,21]. Those with an FPG level >100 mg/dl and < 126 mg/dl were classified as having IFG, and those with a two-hour postchallenge glucose level of 140 mg/dl to < 200 mg/dl were classified as having IGT [20]. We used US census estimates of the 2007 population to scale prevalence to the US population. All of the population surveys used to derive our estimates underwent human subjects review and obtained informed consent on all participants.

On the basis of results of a systematic review showing annualized relative risks for diabetes associated with IFG, IGT, and IFG and IGT combined [22] and on our review of four prospective studies [23-26], we estimated that the initial total diabetes incidence rate (the combined rate for diagnosed and undiagnosed diabetes) in the general US adult population was 1.22% per year. This overall estimate was the weighted average of estimates for adults with normal glucose levels (0.3%), those with IFG (2.4%), and those with IFG and IGT (3.9%). Our estimates of diabetes-related mortality rates were based on population projections from the US Census Bureau and study results showing that the annual relative mortality risk (relative to the risk among people without diabetes) was 1.77 among people with undiagnosed diabetes and 2.11 among those with diagnosed diabetes [27,28].

We projected future diabetes incidence rates in the absence of any intervention using logistic growth curves and Bayesian estimation as described by Boyle et al. (2010) [13]. Primary analyses in this report are based on the lower bound scenario from recently published projections [13], roughly equivalent to a scenario in which the current incidence rate is stabilized at 1.22% per 1,000 per year (hereafter referred to as the “flat trend scenario”). Modeling was done using WinBUGS software [13].

Our projections are based on the following five-state matrix in which we define *N(t)* as a row vector containing the number of people in each of the five states at time *t*. *B(t)* is a row vector of births into each of the states at time *t*. *M(t)* is a row vector of net migration into each of the states. Given initial values *N(2007)* projections are calculated via:

$$N\left(t\right)=N\left(t-1\right)P\left(t\right)+B\left(t\right)+M\left(t\right).$$

When no intervention is applied the model is constrained to be consistent with Census Bureau projections.

We let *P(t)* be a transition matrix of the probabilities of moving to or staying in each of the states at time *t* given the state occupied at time *t-1*. At any given time *P(t)* has the following form:

$$\left[\begin{array}{ccccc} p_{11} & 0 & p_{13} & p_{14} & p_{15}\\ 0 & p_{22} & p_{23} & 0 & p_{25}\\ 0 & p_{31} & p_{33} & 0 & p_{35}\\ 0 & 0 & 0 & p_{44} & p_{45}\\ 0 & 0 & 0 & 0 & p_{55} \end{array}\right].$$

For example, *p*_13_ is the probability of moving to state 3 at time *t* given occupying state 1 at time *t-1*. Entries in the matrix noted 0 are transitions that cannot occur. Note that only the non-zero elements of *P* vary over time. The rows of *P* sum to one minus the death rate for the corresponding state. There were several important assumptions of the model. First, people cannot move from diabetes to no diabetes; this assumption is reasonable because remission is rare [29]. Second, the relative risks of death for the two diabetes states versus the no diabetes state are constant over time; this assumption is more likely to lead to a conservative estimate of the number of future cases, because lower death rates among people with diabetes mean greater prevalence. Third, the assumed transition rates from non-diabetes to diagnosed or undiagnosed diabetes are constant multiples of the transition rate from undiagnosed to diagnosed diabetes. A more detailed description of these models, including all assumptions, references for key parameter estimates, and algebraic derivations, have been previously published [13]. The programs for implementing the models were written in GAUSS [30].

In sensitivity analyses, we also examine the impact of interventions under a scenario of background incidence of the posterior means, roughly equivalent to a continuation of the rate of increase observed over the past decade. Due to model run-time, a full Monte Carlo uncertainty assessment of uncertainty, while desirable, is infeasible. Therefore, we relied on a model emulation approach to approximate the uncertainty in the number of cases presented [31]. Details of the method appear in the Additional file 1. Briefly, we use subjective means to assess uncertainty in intervention effectiveness, due to an absence of data. We treat multiple runs of the model as though they were a sample, fit a linear model to those runs, and use that linear model for error propagation, using our measures of parameter uncertainty. This allows us to create approximate credibility intervals.

## Results

In the absence of any preventive intervention, we projected that 51.7 million new cases of diabetes among US adults would occur by 2030 (Table 1), that the number of US adults with diabetes (diagnosed and undiagnosed) would increase from 27.8 million in 2007 to 60.7 million in 2030, and that the diabetes prevalence rate among US adults would increase from 12.9% to 22.7% during this period.

**Table 1:** Projections of cumulative diabetes incidence, diabetes prevalence, and diabetes prevalence rates through 2030 among US adults, by type of diabetes intervention strategy*

**Intervention strategy**	**2007**	**2010**	**2015**	**2020**	**2025**	**2030**	**Change (2007 to 2030)**	**Relative attenuation (%)**
**None**								
Cumulative diabetes incidence (millions)	2.3	6.8	17.9	29.1	40.3	51.7		
Diabetes prevalence (millions)	27.8	33.1	41.2	48.7	55.3	60.7	32.9	---
Diabetes prevalence rate (%)	12.9	14.8	17.5	19.7	21.5	22.7	9.8	---
**High-risk strategy (lifestyle intervention for adults with IFG and IGT)**								
Cumulative diabetes incidence (millions)	2.2	6.5	17.3	28.1	39.0	50.1		
Diabetes prevalence (millions)	27.8	32.8	40.6	47.9	54.2	59.5	31.7	−3.6
Diabetes prevalence (%)	12.9	14.7	17.2	19.3	21.0	22.2	9.3	−5.1
**Moderate-risk strategy (lifestyle intervention for adults with IFG)**								
Cumulative diabetes incidence (millions)	2.1	6.2	16.4	26.7	37.1	47.7		
Diabetes prevalence (millions)	27.8	32.5	39.8	46.6	52.6	57.6	29.8	−9.4
Diabetes prevalence rate (%)	12.9	14.5	16.9	18.8	20.3	21.5	8.6	−12.2
**Population strategy (risk reduction policies targeting entire population)**								
Cumulative diabetes incidence (millions)	2.2	6.7	17.6	28.5	39.6	50.8		
Diabetes prevalence (millions)	27.8	33.0	40.9	48.2	54.7	60.0	32.2	−2.1
Diabetes prevalence rate (%)	12.9	14.7	17.3	19.5	21.2	22.4	9.5	−3.1
**Combination of moderate-risk and population strategies**								
Cumulative diabetes incidence (millions)	2.0	6.1	16.1	26.3	36.7	47.1		
Diabetes prevalence (millions)	27.8	32.4	39.6	46.3	52.2	57.1	29.3	−10.9
Diabetes prevalence rate (%)	12.9	14.5	16.8	18.7	20.2	21.3	8.4	−14.3

*The high-risk strategies are assumed to result in a net 12.5% reduction in diabetes incidence in the 8.3% of the population who have both IFG and IGT. The moderate-risk strategy is assumed to result in a 12.5% reduction in the 26.7% of the population who have either IFG or IGT. The population strategy is assumed to result in a net 2% reduction in diabetes incidence in the entire population. The combined strategy results in a 12.5% reduction in those with IFG or IGT and a 2% reduced incidence in diabetes in the rest of the population.

**Figure 1:**
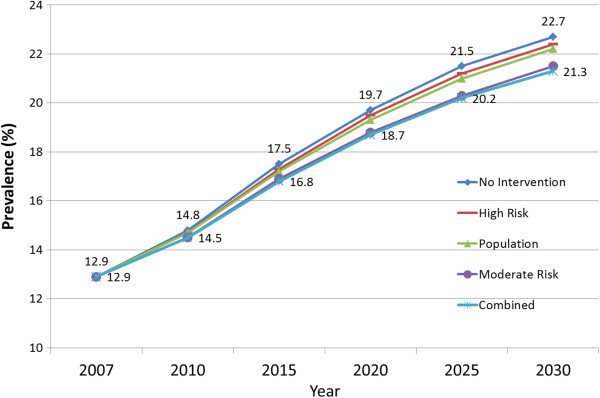
Projected diabetes prevalence rates among US adults according to five prevention policy scenarios, 2007 to 2030.

We projected that the combined strategy would be the most effective and that its implementation would result in 4.6 million fewer new diabetes cases through 2030, reduce the prevalence of diabetes in 2030 by 3.6 million cases, and reduce the diabetes prevalence rate in 2030 from 22.7% to 21.3%. Our other projections indicated that the moderate-risk approach (intervention for the 26.7% of the population with IFG) would result in 4.0 million fewer new diabetes cases over 20 years, reduce the diabetes prevalence in 2030 by 3.1 million cases, and reduce the diabetes prevalence rate in 2030 from 22.7% to 21.5%. The high-risk approach (intervention for the 8.3% of the population with both IFG and IGT) would reduce the projected 2030 prevalence of diabetes by 1.2 million cases and reduce the projected prevalence rate in 2030 from 22.7% to 22.2%. The population-wide approach would have a projected impact similar to the high-risk approach, reducing 0.9 million cumulative cases, 0.7 million prevalent cases in the year 2030, and reducing the prevalence rate in 2030 from 22.7% to 22.4% (Table 1; Figure 1). Even if the most effective strategy is implemented (the combined strategy), our projections indicate that the diabetes prevalence rate would increase by about 65% over the 20 years (i.e., from 12.9% in 2010 to 21.3% in 2030). Compared with the projected increase in diabetes prevalence in the absence of any national intervention, these estimates indicate that the relative attenuation in the increase in prevalence would be 5.1%, 12.2%, 3.1%, and 14.3% with implementation of the high-risk, moderate-risk, population, and combined scenarios, respectively.

**Figure 2:**
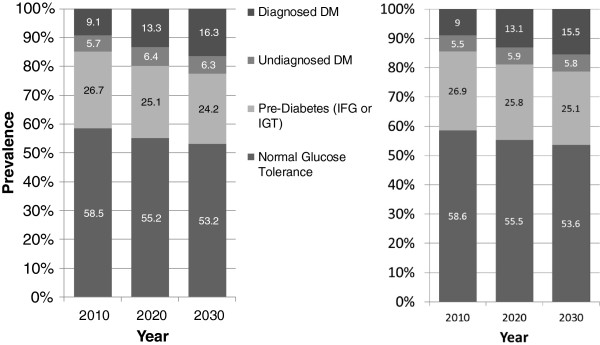
Projected prevalence rates of normal glucose tolerance, prediabetes, undiagnosed diabetes, and diagnosed diabetes among US adults from 2010 through 2030 if no national diabetes prevention intervention is implemented and if the combined intervention is implemented.

We also projected that implementation of the combined intervention would reduce the diagnosed diabetes prevalence rate in 2030 by 0.8 percentage points (from 16.3% to 15.5%), reduce the undiagnosed diabetes prevalence rate by 0.5 percentage points, but increase the predicted pre-diabetes prevalence rate by 0.9 percentage points (Figure 2). The projected lower prevalence of persons with normal glucose levels (low risk) and with prediabetes occurred because the incidence of diabetes was higher, which shifted persons out of the low-risk and prediabetes categories.

In a one-variable-at-a-time sensitivity analysis, in which we assumed that the diabetes incidence rate among US adults would continue to increase according to recent trends (roughly 60% increase over the past 20 years, i.e., the middle range scenario described by Boyle et al., [11]), we found that the relative benefits of the alternative prevention strategies were similar to what we projected them to be in our base case analyses (Table 2). However, if assumed that the structured lifestyle intervention would reduce the incidence rate in the target population by 6.25% rather than 12.5%, or that the population-wide intervention would reduce the population incidence rate by 4% rather than 2%, then the population-wide approach would be slightly more beneficial than the high-risk approach. However, even under this optimistic assumption about the effectiveness of the population-wide approach, we projected that its implementation would reduce the number of diabetes cases by only about half as much as the moderate-risk approach and only about a third as much as the combined approach.

**Table 2:** Results of one-variable-at-a-time sensitivity analyses showing effects of changes in two base case assumptions on the projected reduction in diabetes prevalence among US adults in 2030 following implementation of four diabetes prevention strategies

**Assumptions**	**Projected reduction in diabetes prevalence compared to no intervention**			
	**Moderate-risk [millions]**	**High-risk [millions]**	**Population [millions]**	**Combined [millions]**
**Base case assumptions:**				
Flat background diabetes incidence rate*	1.2 [3.1]	0.5 [1.2]	0.3 [0.7]	1.4 [3.6]
Lifestyle intervention will reduce yearly diabetes incidence rate by 12.5% in target population				
Population-wide intervention will reduce the yearly diabetes incidence rate by 2% among all US adults				
**Adjustments to base case assumptions**				
Increasing background incidence rate*	1.4 [3.7]	0.5 [1.4]	0.3 [0.8]	1.6 [4.2]
Lifestyle intervention will reduce yearly diabetes incidence rate by 25% in target population	2.5 [6.4]	1.0 [2.6]	0.3 [0.7]	2.7 [6.9]
Lifestyle intervention will reduce yearly diabetes incidence rate by 6.25% in target population	0.6 [1.5]	0.3 [0.6]	0.3 [0.7]	0.8 [2.0]
Population-wide intervention will reduce yearly diabetes incidence rate by 1% among all US adults	1.2 [3.1]	0.5 [1.2]	0.2 [0.3]	1.1 [2.8]
Population-wide intervention will reduce yearly diabetes incidence rate by 4% among all US adults	1.2 [3.1]	0.5 [1.2]	0.6 [1.4]	1.6 [4.0]

*Flat background diabetes incidence estimated at 1.22% per year. Increasing background diabetes incidence rate estimated based on the middle incidence projection from Boyle et al., wherein incidence increases by 75% between 2007 and 2050.

Finally, the results of our error propagation analysis appear in Table 3. The intervals are relatively wide, due to the substantial uncertainty in the effectiveness of a hypothetical intervention, particularly a population-wide intervention. However, even with the large uncertainty the combined scenarios would result in a net reduction in diabetes prevalence over what would occur with no intervention, but diabetes prevalence would still increase over its current level.

**Table 3:** Uncertainty for the projected reduction in diabetes prevalence for four intervention scenarios

**Intervention (r**^**2 **^**for linear model approximation)**	**95% approximate credibility interval for projected reduction in diabetes prevalence, compared to no intervention (millions)**
Combined (0.92)	0.9 to 6.3
Population (0.93)	0.0 to 0.9^1^
High-risk (0.91)	0.0 to 3.0^1^
Moderate-risk (0.93)	0.1 to 5.9

^1^Due to their approximate nature, the calculated lower bounds of these intervals were small negative numbers. Since it is not plausible that interventions could increase prevalence, we have truncated these intervals at 0.0.

## Discussion

Results of several controlled trials have shown that lifestyle interventions can reduce the incidence of type 2 diabetes among people at high risk [7-9,16,32]. Questions remain, however, about the impact of these targeted interventions, as well as those of population-wide interventions, on future diabetes incidence and prevalence rates. Although drawing definitive conclusions is difficult, due to parameter uncertainty, our model suggested that the greatest reduction in the number of diabetes cases would be achieved through implementation of a multitiered strategy involving a structured lifestyle intervention for adults with IFG in conjunction with risk-reduction policies aimed at the entire population. We projected that such an approach would result in 4.6 million fewer diabetes cases over 20 years and 3.6 million (14%) fewer Americans with diabetes in 2030 than corresponding projections based on the assumption of no national intervention. These projected reductions are substantial given the high lifetime risk of amputation, kidney disease, and CVD and the extensive lifetime health care costs among people with diagnosed diabetes [33,34]. We projected that the next most effective strategy—and possibly the most plausible one under a setting of limited resources—would be the moderate-risk strategy (a lifestyle intervention for about 27% of the population), which we projected would result in a 12.2% attenuation in what we projected the increase in diabetes prevalence would be in the absence of any additional diabetes prevention intervention. We projected that the high-risk approach would produce a prevalence reduction only about one-third as large as the combined approach and that the population-wide approach would produce a prevalence reduction only about one-fourth as large; for both of these scenarios, our error propagation analysis reminds us that even these gains are somewhat uncertain. Our projection that the population-wide approach, by itself, would be relatively ineffective was in part because our assumption about the risk reduction to be derived was lower, but also because most people receiving the intervention would not have developed diabetes anyway.

Despite the benefits that can be achieved with preventive interventions, particularly the combined and moderate-risk approaches, our projections indicate a need to develop new options for diabetes prevention and/or increase the reach of both primary and secondary prevention efforts. We projected that the diabetes prevalence rate in the United States will surpass 20% by 2030 even if the combined strategy is implemented; a large increase in prevalence will occur even at the upper credibility bound for effectiveness of this intervention, the most optimistic plausible case. This projected increase was based on data showing a steady increase in the US diabetes incidence rate over the past 20 years [35,36], a decline in diabetes mortality rates as diabetes has become increasingly treatable while remaining largely incurable [4,37], and transition of the baby boom generation into the age range corresponding to peak diabetes incidence [38].

Our projection that the US diabetes prevalence rate will increase substantially even under the combined intervention scenario indicates that the success of currently known primary prevention efforts will not diminish the need for more effective programs to help people manage their diabetes and prevent its complications. Diabetes has diffuse effects, leading to multiple microvascular and macrovascular complications and an increased risk for aging-related disability. Because numerous clinical, screening, community, and educational interventions reduce the incidence of complications [39,40], many diabetes experts have argued that delivery of proven interventions is more important for the diabetic population than the development of new therapies [41]. Our findings similarly indicate the importance of timely and efficient screening and treatment for hypertension, hyperlipidemia, chronic kidney disease, and diabetic eye and foot disease among people with diagnosed diabetes, especially as people with diabetes live longer with the condition. We should remember, however, that such success in the reduction of diabetes complications will likely lead to lower diabetes mortality rates and thus can contribute to an increase in diabetes prevalence.

Our projections are limited by our assumptions. If the US diabetes incidence rate continues to increase at its recent pace (rather than stabilizing at the 2007 rate, as we assumed in our primary analyses) and all other assumptions are unchanged, the prevalence of diabetes in 2030 would be three to four percentage points higher than we projected [13]. If mortality rates associated with diabetes decline over time, as suggested by the results of some studies [4,42], and all other assumptions are unchanged, the prevalence of diabetes in 2030 would also be higher than we projected. Our conclusions about the relative impact of various intervention policies on future diabetes prevalence rates would be relatively unaffected by changes in our assumptions about future diabetes incidence rates. However, our model also reflected our assumption that the interventions would have no effect on diabetes mortality rates other than their effect on diabetes risk. Any additional reductive effect on mortality rates associated with the interventions would actually result in smaller differences in diabetes prevalence projections among the intervention scenarios than those we reported. Perhaps the biggest practical limitation of our model is that it did not allow us to project diabetes prevalence by age, sex, or racial/ethnic group. Because the absolute prevalence of diabetes is much higher and more influenced by mortality rates among older adults than among younger adults, interventions could be more effective in reducing future diabetes prevalence rates among younger adults than our projections indicated they would be for the overall US adult population. Similarly, the prevalence rates among younger adults could stabilize or even decrease even though the prevalence rate among all US adults continues to rise.

As indicated by both our one-variable-at-a-time and error propagation analyses, our findings are sensitive to our assumptions about the effectiveness of interventions. Results of several randomized controlled trials among people with prediabetes have found a 50% or more reduction in incidence among people with prediabetes, with some finding extended effects up to 20 years later [9,16,32,43]. Similar levels of weight loss among people with prediabetes as those seen in the diabetes prevention trials have been observed in interventions provided in community settings with economically sustainable staff and facilities [44-46]. In the absence of data that would support a more complicated assumption, we made the simple assumption that the relative risk reduction associated with lifestyle intervention was the same in people with IFG as those with both IFG and IGT. However, our modeling did account for differences in the absolute risk between risk strata that would, in turn, lead to different levels of absolute risk reduction in response to an intervention. The accuracy of our projections was also dependent on the extent to which the “rule of halves” that has been reported in hypertension studies, wherein about half of the target population would be identified and referred to a lifestyle program and about half of those referred would actually initiate participation in a lifestyle program, is also applicable to diabetes lifestyle interventions [14]. However, even if these assumptions are correct, the moderate- and high-risk approaches would require a strong system of reimbursement for providers of community lifestyle programs that does not yet exist.

The impact of a population-wide approach to diabetes prevention is particularly hard to predict because there have been few estimates of the health impact of such an approach; this is reflected in the large uncertainty in this term in our error propagation analysis. We based our estimates that population-wide strategies would lead to a 1 to 4% reduction in the relative incidence of diabetes on the results of studies concerning community-based efforts to reduce rates of CVD [17,47]. Given the strong relationship between obesity and diabetes, the success of a population-wide approach to diabetes prevention will ultimately depend on the extent to which such an approach is successful in reducing obesity rates [48]. Proposed approaches to doing this include taxing sugared beverages or sugar itself, mandating wide-scale menu labeling, providing incentives to increase the availability of healthy foods, encouraging urban designs that promote physical activity, and enhancing awareness of and education about risk factors and prevention behaviors [49-51]. Programs to enhance employer-based health-promotion programs also may help reduce diabetes rates, particularly if they can effectively stratify the population by levels of diabetes risk and the intensity of the intervention appropriate for that risk level [17,49-51]. Since these interventions could have important health effects on additional chronic conditions such as hypertension, cardiovascular disease, and disability, the pure focus of our analysis on prevention of diabetes cases may not reflect the value or the differential impact of the hypothetical interventions. Finally, the feasibility of any intervention depends on both effectiveness and cost. Real interventions similar to the hypothetical ones described here could have vastly different costs. However, due to the structure of our model, the scarcity of the literature, and the lack of details as to how national interventions like the ones we describe might be implemented, it was not feasible for us to consider cost in our estimates.

Although our projections of future diabetes prevalence rates among US adults are based on numerous assumptions, they are likely more accurate than previous projections because they are based on more recent data, including updated projections of future diabetes incidence rates and updated population projections. Most importantly, because the model we used estimated diabetes incidence and mortality rates for people at different stages of diabetes risk and in different diagnostic categories, it allowed us to more accurately project the impact of various intervention strategies. However, our projections, and others like these, need to be continuously updated as new intervention effectiveness and epidemiologic data become available. We hope that more rigorous controlled studies or perhaps natural experiments of population-targeted approaches for diabetes prevention will emerge in the future to permit more confident modeling of the impact on future prevalence.

The results of our analyses indicate a need for the provision of effective lifestyle interventions to the large number of adults with prediabetes as well as support research aimed at improving the effectiveness of these interventions. They also suggest a need for research designed to improve the effectiveness of population-wide approaches to diabetes prevention. Unless both strategies are much more effective than we assumed they will be in our analyses, the prevalence of diabetes among US adults will continue to increase even if the US health care system adopts something like the combined diabetes prevention strategy that we described. This continued increase in diabetes prevalence will result in an increase in demands for diabetes management and treatment, including services related to the prevention of diabetes complications and diabetes-related disability, and these services will need to be strengthened along with primary diabetes prevention efforts.

The findings and conclusions in this report are those of the authors and do not necessarily represent the official positions of the Centers for Disease Control and Prevention.

## Competing interests

The authors declare that they have no competing interests.

## Authors’ contributions

EWG and DFW conceptualized and supervised the study and drafted the manuscript. JPB, LEB, and TJT developed the statistical models and conducted statistical analyses. ALA contributed to writing and critical review of the manuscript. All authors read and approved the final manuscript.

## Supplementary Material

Additional file 1 **Details of error propagation model. Table S1.** Parameters used in error propogation model, including change in projected reduction in diabetes prevalence (Y), natural logarithm of magnitude of effectiveness of targeted intervention (X_1_), and natural logarithm of population-wide intervention effectiveness (X_2_). **Table S2.** Click here for file
