# Supplementary material for: Modeling the impact of prevention policies on future diabetes prevalence in the United States: 2010–2030
Source: Popul Health Metr. 2013 Sep 18;11:18. doi: 10.1186/1478-7954-11-18 (PMC3853008; doi:10.1186/1478-7954-11-18)
Supplement: Additional file 1 — Details of error propagation model. Table S1. Parameters used in error propogation model, including change in projected reduction in diabetes prevalence (Y), natural logarithm of magnitude of effectiveness of targeted intervention (X1), and natural logarithm of population-wide intervention effectiveness (X2). Table S2. [file 1478-7954-11-18-S1.doc]

Additional file 1

Details of error propagation model

Let Y denote change in projected reduction in diabetes prevalence compared to no intervention (millions). Let X_1_ denote the natural logarithm of the amount by which the lifestyle intervention will reduce diabetes incidence in the target population (effectiveness). Let *X*_2_ denote the natural logarithm of the population-wide intervention’s effectiveness. Assume all other parameters are known and set to the base case; although there is uncertainty associated with all parameters, these parameter values are the most controversial, and therefore the focus of our analysis. We used the logarithmic transform to make parameter estimates uncorrelated. For example, Table 2 provides the data (combined scenario) (Table S1):

**Table S1 Parameters used in error propogation model, including change in projected reduction in diabetes prevalence (Y), natural logarithm of magnitude of effectiveness of targeted intervention (X_1_), and natural logarithm of population-wide intervention effectiveness (X_2_)**

| **Y** | **X_1_** | ***X*_2_** |
| --- | --- | --- |
| 1.40 | 2.53 | .69 |
| 2.70 | 3.22 | .69 |
| .80 | 1.83 | .69 |
| 1.10 | 2.53 | .00 |
| 1.60 | 2.53 | 1.39 |

Using SPSS Version 19, we fit the model

$$Y=\beta_{0}+\beta_{1}X_{1}+\beta_{2}X_{2}+\varepsilon$$

where

$\varepsilon\sim N\left(O,\sigma^{2}\right).$

This model yields an r^2^ of 0.916 (Table S2).

**Table S2**

| **Model** | | **Unstandardized Coefficients** | |
| --- | --- | --- | --- |
|  |  | **B** | **Std. Error** |
| 1 | (Constant) | −2.193 | .808 |
|  | X_1_ | 1.371 | .304 |
|  | *X*_2_ | .361 | .304 |

with $\hat{\sigma}=0.298.$

Then if X_1_ and *X*_2_ are random, substituting maximum likelihood estimates for parameter values, we obtain

$$Var\left(\hat{Y}\right)=Var\left(\hat{\beta}_{0}\right)+Var\left(X_{1}\hat{\beta}_{1}\right)+Var\left(X_{2}\hat{\beta}_{2}\right).$$

If U and V are uncorrelated random variables with finite variances, then it can readily be established that Var(UV) = Var(U) (EV)^2^ + Var(V)(EU)^2^ + Var(U)Var(V). Assuming the effectivenesses of the interventions are uncorrelated and applying this, we get

$$Var\left(\hat{Y}\right)=Var\left(\hat{\beta}_{0}\right)+Var\left(X_{1}\right)\left(\hat{\beta}_{1}\right)^{2}+Var\left(\hat{\beta}_{1}\right)\left(EX_{1}\right)^{2}+Var\left(X_{1}\right)Var\left(\hat{\beta}_{1}\right)+Var\left(X_{2}\right)\left(\hat{\beta}_{2}\right)^{2}+Var\left(\hat{\beta}_{2}\right)\left(EX_{2}\right)^{2}+Var\left(X_{2}\right)Var\left(\hat{\beta}_{2}\right).$$

Using the above equation, plugging in sample estimates for the means and variances of the estimates of the beta coefficients the base-case values for X_1_ and *X*_2_, we can produce an estimate:

$$Var\left(\hat{Y}\right)\approx\left(.808\right)^{2}+Var\left(X_{1}\right)\left(1.371\right)^{2}+\left(.304\right)^{2}\left(2.53\right)^{2}+Var\left(X1\right)\left(.304\right)^{2}+Var\left(X_{2}\right)\left(.361\right)^{2}+\left(.304\right)^{2}\left(.69\right)^{2}+Var\left(X_{2}\right)\left(.304\right)^{2}$$

which simplifies to

$$Var\left(\hat{Y}\right)\approx\left(.808\right)^{2}+\left(.304\right)^{2}\left(2.53\right)^{2}+\left(.304\right)^{2}\left(.690\right)^{2}+Var\left(X_{1}\right)\left\{\left(1.371\right)^{2}+\left(.304\right)^{2}\right\}+Var\left(X_{2}\right)\left\{\left(.361\right)^{2}+\left(.304\right)^{2}\right\}=1.29+1.97 Var\left(X_{1}\right)+0.22 Var\left(X_{2}\right).$$

Thus, we have an approximation to $Var\left(\hat{Y}\right)$ if all parameters other than the effectiveness of the two interventions are assumed known, if we assume that our linear model is an adequate fit, and if we can assume reasonable values for Var(X_1_) and Var(*X*_2_). Similar methods (differing in that uncertainty about irrelevant parameters is not considered, such as the effectiveness of a targeted intervention in the population-wide intervention) are applied to all intervention scenarios, and details are not presented.

Hard data on the uncertainty in effectiveness are unavailable, due to these national-level interventions never having been implemented. Therefore, we used estimates based on what data are available and our own best judgment. Effectiveness for a targeted intervention, based on data from the Diabetes Prevention Program, is relatively well understood. Therefore, we consider the range (half the base case) to (twice the base case) to be a 90% credible interval (standard error for natural logarithm of effectiveness = 0.42). Effectiveness for a population-wide intervention is much more speculative. Therefore, we consider the range (half the base case) to (twice the base case) to be a 50% credible interval (standard error for natural logarithm of effectiveness = 1.07). We construct 95% credibility intervals for the projected reduction in diabetes prevalence.

Finally, we also considered models in which the reduction in number of cases was transformed. For example, had we modeled the natural logarithm of the decrease in the number of cases, then a negative lower bound would have been impossible. However, models with transformed dependent variables were both more complicated than the model we presented and fit the data no better. Therefore, we present the untransformed case.
